# Sustained Attention is Associated with Error Processing Impairment: Evidence from Mental Fatigue Study in Four-Choice Reaction Time Task

**DOI:** 10.1371/journal.pone.0117837

**Published:** 2015-03-10

**Authors:** Yi Xiao, Feng Ma, Yixuan Lv, Gui Cai, Peng Teng, FengGang Xu, Shanguang Chen

**Affiliations:** 1 National Key Laboratory of Human Factors Engineering, China Astronaut Research and Training Center, Beijing, China; 2 School of Aerospace, Tsinghua University, Beijing, China; 3 Department of Ergonomics, School of Biological Science and Medical Engineering, Beihang University(BUAA), Beijing, China; Beijing Normal University, Beijing, CHINA

## Abstract

Attention is important in error processing. Few studies have examined the link between sustained attention and error processing. In this study, we examined how error-related negativity (ERN) of a four-choice reaction time task was reduced in the mental fatigue condition and investigated the role of sustained attention in error processing. Forty-one recruited participants were divided into two groups. In the fatigue experiment group, 20 subjects performed a fatigue experiment and an additional continuous psychomotor vigilance test (PVT) for 1 h. In the normal experiment group, 21 subjects only performed the normal experimental procedures without the PVT test. Fatigue and sustained attention states were assessed with a questionnaire. Event-related potential results showed that ERN (p < 0.005) and peak (p < 0.05) mean amplitudes decreased in the fatigue experiment. ERN amplitudes were significantly associated with the attention and fatigue states in electrodes Fz, FC1, Cz, and FC2. These findings indicated that sustained attention was related to error processing and that decreased attention is likely the cause of error processing impairment.

## Introduction

Human errors exist almost pervasively in the majority of tasks, which need cognition and manipulation, and may possibly cause vital negative effects, especially in such conditions as operating machines, driving vehicles, and extra-vehicular activities. Many human error accidents occur in human space flight [1]. Error processing constitutes a chain of dynamic neurocognitive processes including error monitoring and behavioral adjustment and involving continuous checking of ongoing actions and mobilization of cognitive control and corrective action [2,3]. Thus, error processing has significance in individual adaptation after an error, which might be considered as an index to assess the corrective action in a special task, such as rendezvous and docking in human space flights. Investigating the cognitive mechanism of error processing for its potential application in appropriate strategies to resolve errors is significant. Although numerous studies have been amassed on the cognitive mechanism of error processing, few studies have examined the influence of sustained attention on event-related potential (ERP) indices of error processing. The link between sustained attention and error processing, especially the quantitative relationship, might be an important theory foundation for personnel selection and training of specific positions, such as pilots and astronauts, in which human error is unacceptable. To a certain extent, error processing can be used to predict sustained attention based on the quantitative relationship.

Error processing was investigated with regard to its correlation with ERPs, such as error-related negativity (ERN) and error positivity (Pe) [2,4–8]. ERN is a negative deflection in the ERP with approximate peaks from 50 ms to 100 ms after an incorrect response, which reaches its maximum at fronto-central scalp locations. Correct-related negativity (CRN) resembles ERN in latency, but usually has a smaller amplitude in the correct response [9–11]. Pe is a positive centro-parietal deflection occurring approximately 200 ms to 400 ms after erroneous responses [4,6,12].

Numerous studies have investigated ERN of issues, such as the relationship between attention and ERN amplitude changes based on theory analysis, the relationship between error salience and ERN amplitude changes, and the relationship between effective state and ERN amplitude changes. However, the quantitative relationship between attention and ERN amplitude under the mental fatigue state has been seldom investigated. Moreover, how ERN changes in the fatigue state remains unclear. Attention is highly important in error processing as a central cognitive character. Conflict monitoring theory indicated that increased attention to task-relevant information resulted in enhanced ERN amplitude, whereas decreased attention to task-relevant information resulted in diminished ERN amplitudes [13–16]. Meanwhile, the dual-task design resulted in diminished ERN amplitudes [8,17]. ERN amplitudes also diminished when less attention resources were available for the primary task [18–22]. However, previous studies focused only on the theoretical analysis of the relationship between attention and ERN amplitudes, without quantitative analysis of correlation and appropriate assessment of the attention state. Moreover, the definite type of attention related to ERN has also not been clearly presented. As such, the relationship between attention and ERN amplitudes remains debatable and unclear. How fatigue level may reduce ERN amplitudes remains unknown. The role of attention in ERN amplitude change is still unclear. Boksem et al. [20] conducted a 2 h performance task to induce fatigue and then finished a 20 min flanker task. Results showed that ERN amplitudes and attention decreased, resulting in failure to monitor the error response. However, in the studies of Clayson et al. and Wiswede et al. [14,23], a lower fatigue level was induced during the flanker task, which showed that ERN amplitudes may decrease or remain unchanged in the fatigue state. Different results of these studies may be caused by different fatigue levels. Attention was presented as one factor that can likely influence ERN amplitudes in mental fatigue based on theory analysis. However, a quantitative description of the relationship between attention and ERN was not provided in previous studies.

A variety of tasks, including the flanker, Go/NoGo, Stroop, SART, and two-choice reaction time (RT) tasks, were conducted in ERN research [2,24–28]. Whether ERN can be observed in different tasks, such as the four-choice RT task; how ERN amplitude changes in the fatigue state; and the quantitative relationship between sustained attention and ERN amplitudes were examined in this study. First, a 1 h psychomotor vigilance test (PVT) task was used to induce mental fatigue for the fatigue group. Then, the four-choice RT task was used for the two groups to evoke the error-related ERPs. The changes of ERN and Pe and the relationship between sustained attention and error processing in the two groups were investigated.

## Materials and Methods

### Participants

Forty-one male volunteers from the China Astronaut Research and Training Center and BeiHang University participated in the study. The volunteers were 19 years to 34 years of age, with an average of 26 years. The volunteers were all postgraduate or graduate students, except one junior student. All participants were right-handed and had normal or corrected-to-normal vision, without a history of psychiatric disorders. The participants were randomly divided into two groups based on age. This research was conducted in accordance with the principles of the Declaration of Helsinki. The ethics committee of the China Astronaut Research and Training Center approved this study. All participants provided a written informed consent prior to the experiments and received adequate remuneration at the end of this study.

Participants were divided into two groups, namely, the normal experiment group (n = 21) and the fatigue experiment group (n = 20). Based on the criteria of Grutzmann et al. that ERN cannot be extracted from fewer than five errors, six participants (n = 1, data acquisition artifacts; n = 5, fewer than five errors) and five participants (n = 2, data acquisition artifacts; n = 3, fewer than five errors) were excluded from further analysis in the normal and fatigue experiment groups, respectively [20,23,29,30].

## Task and Procedures

### Task to Induce Fatigue

**Fig 1:**
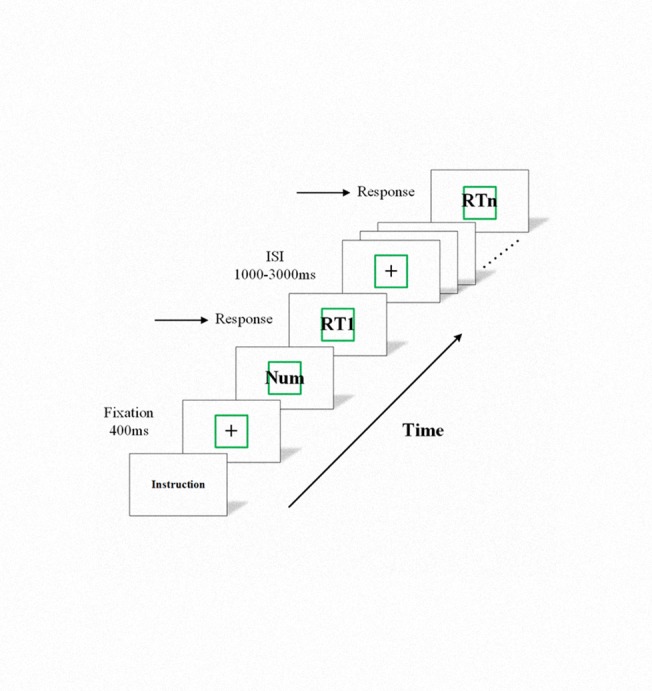
PVT procedures. A fixation point was first presented for 400 ms. Then, each Arabic numeral was presented for 1,000 ms. Participants were asked to press the space key as soon as possible. The ISI was 1,000 ms to 3,000 ms.

The 1 h visual PVT was used to induce fatigue. The fatigue levels were between the concepts of Boksem et al., Claysonet al., and Wiswede et al. in theory [20,23,29,30]. The PVT procedures were shown in Fig. 1.

Stimuli were displayed in white against a black background on a 24 in. computer liquid crystal display (LCD) monitor (refresh rate of 60 Hz). At the beginning of each trial, participants see a fixation mark at the center of the screen for 400 ms. Afterward, Arabic numerals in a green box appear and are displayed for 1,000 ms or disappear if the participant responded. After 1,000 ms to 3,000 ms (inter stimulus interval [ISI]), a new trial begins. Stimuli were varied pseudo-randomly across trials. The subjects were instructed to respond as quickly and accurately as possible by pressing the space key. However, when no numeral was displayed, no response was expected. The subjects underwent a total of 60 min of PVT test. A 1 min rest period in half an hour was provided.

### Four-Choice Reaction Time Task

Although used for children or patients with obsessive–compulsive symptoms, the two-choice RT tasks may not be difficult enough for the subjects, namely, university students, to induce sufficient error times for ERN [24,27]. We selected the four-choice RT tasks because the error times in the four-choice RT tasks were observed to reach the levels sufficient for ERN extraction within the specified time in normal conditions.

**Fig 2:**
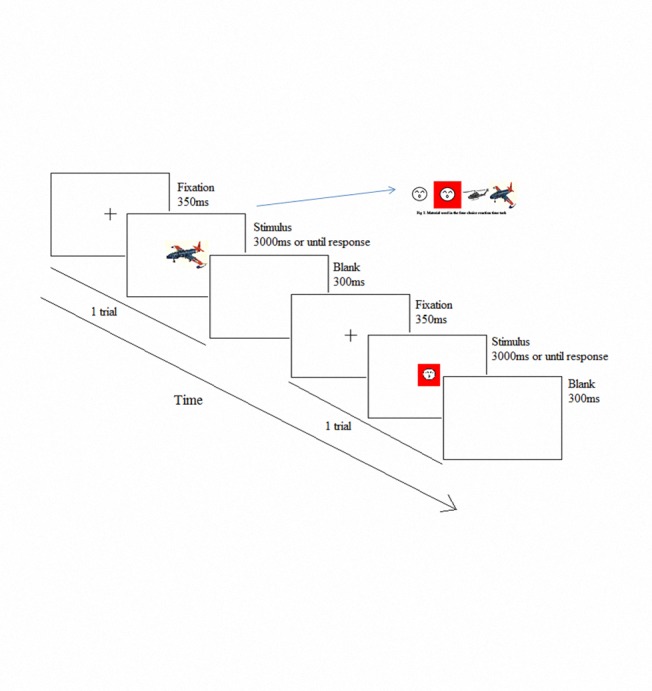
Four-choice RT task procedures. First, a fixation point was presented for 350 ms. Then, each stimulus was presented for 3,000 ms. Participants were required to press the keys as soon as possible. The minimum ISI was 300 ms.

Stimuli were displayed in white against a black background on a 24 in. LCD (refresh rate of 60 Hz). At the beginning of each trial, participants see a fixation mark at the center of the screen for 350 ms. Afterward, a picture (e.g., a plane, helicopter, red face, or white face; the materials are shown in Fig. 2) appears and is displayed for 3,000 ms or disappears if the participant responded. After at least 300 ms (ISI; if the RT is within 3,000 ms, the ISI = (3,000 − Acc + 300) ms), a new trial begins. Participants were instructed to respond as quickly and accurately as possible. Participants were required to make key responses by pressing the “J” key when they saw a picture of a plane, pressing the “F” key when they saw a picture of a helicopter, pressing the “U” key when they saw a picture of a red face, and pressing the “R” key when they saw a picture of a white face. The rate for the plane and helicopter was 40% each, whereas the rate for the red face and white face was 10% each. The total number of trials for the RT task was almost 700 in each experiment.

### Fatigue Questionnaire

Participants were administered the fatigue questionnaire, which was developed by the Japanese Association for Industrial Health in 1971 [31], to assess the fatigue and attention states under two conditions. The questionnaire was a four-item self-report inventory designed to measure transient or fluctuating affecting states on four different scales, namely, mental clarity, attention concentration, sleepiness, and comprehensive assessment of fatigue (fatigue subscale). Participants were required to describe and score their feelings on a scale ranging from 1 (very less) to 10 (extremely). In the fatigue questionnaire, a high sleepiness score indicates a higher fatigue level, a high fatigue score indicates a higher fatigue level, a high total score indicates a higher fatigue level, a higher mental clarity score indicates a good mind state but lower fatigue level, and a high attention concentration score indicates a good sustained attention state.

### Procedures

Recruitment materials provided a general description of the study, but did not explain its purpose. No participants discontinued their participation after informed consent was obtained.

After placement of the electroencephalogram (EEG) electrodes, participants were seated 80 cm to 100 cm in front of an LCD monitor and given detailed task instructions. Each participant was asked to attend a practice before the formal experiment to ensure that everyone was familiar with the tasks. In the normal experiment, the questionnaire was recorded before the four-choice RT task. By contrast, in the fatigue experiment, the questionnaire was recorded between the 1 h PVT task and four-choice RT task.

## Statistical Analysis of Behavior Data

Error rates and correct RTs of standard (picture of a plane or helicopter) and deviant (picture of a red face or white face) stimuli were analyzed using the two-tailed t test. Considering that few errors were committed in deviant stimuli trials, RTs computed from such few trials were invalid estimates. Error RT was only analyzed for standard stimuli trials. The RT of post-errors (or post-correct) was computed separately for correct responses preceded by incorrect and correct responses in the standard and deviant conditions. Post-error and post-correct response RTs were compared in the two experiments separately Post-error and post-correct response RTs and post-error slowing (post-error slowing = mean (RT post-error) − mean (RT post-correct)) of the two experiments were compared. The correlation coefficients of post-error (or post-correct) and error-related ERPs based on Spearman correlations were evaluated.

### Statistical Analysis of Fatigue Questionnaire Data

The total fatigue score was calculated for all four subscales. The attention concentration subscale and the other three subscales were also calculated separately. In addition, two-tailed t test was used to analyze the two conditions (normal and fatigue). Spearman correlations between the questionnaire and error-related ERPs were acquired.

### Statistical Analysis of EEG and ERP Data

The EEG and electrooculography data were obtained from 63 sintered Ag-AgCl electrodes mounted in an elastic cap (EasyCap, Brain Products GmbH) with a standard 10/20 system layout. The ground electrode was positioned at AFz and the reference electrode was positioned at FCz on the forehead. Electrode impedances were kept below 5 kΩ. Signals were recorded using the BrainVision Recorder (Brain Products GmbH) with an online low-pass filter (250 Hz). When an electrode reached 70% saturation, a direct current reset was applied. Sampling rate was 1,000 Hz and signals were amplified in the range of ±3.27 mV at a resolution of 0.1 μV.

The EEG data were processed offline using the BrainVision Analyzer 2.0. Software (Brain Products GmbH). Tp9 and Tp10 were selected as the new reference (Brain Products GmbH). Then, EEG data were filtered using a band-pass filter from 0.01 Hz to 100 Hz. After filtration, the semiautomatic inspection method was implemented to inspect raw data. The gradient criterion was 50 μV/ms. The maximum absolute difference allowed was 200 μV and the interval length was 200 ms. Amplitude was between −200 μV and 200 μV. The lowest allowed activity was 0.5 μV. After raw data inspection, the EEG signals were corrected for eye movement artifacts using the artifact rejection method based on Gratton and Coles’ algorithm, which was implemented in the BrainVision Analyzer 2.0 Software [32]. The continuous EEG signals were filtered using a band-pass filter from 0.01 Hz to 35 Hz. Response-locked epochs with a length of 1,200 ms, including a 400 ms pre-response interval, were extracted. The trials with RT between 200 ms and 1,500 ms were used for ERP extraction. Response-locked amplitude averages were computed separately for each participant and each condition. Response-locked ERPs were baseline corrected for the interval from −400 ms to 200 ms prior to the response. ERN and CRN were quantified as the mean and maximum amplitudes from 0 ms to 100 ms post-response. The Pe was quantified as the mean amplitude between the time window of 200 ms and 400 ms following the response. ERN, CRN, and Pe amplitudes were calculated at Fz, Cz, FC1, and FC2. Grand averages were filtered using a low-pass filter of 15 Hz for visual presentation.

The variance of ERN peak amplitudes in the two experiments were normally distributed, but were not homogenous (p < 0.05). The same was true for ERN mean amplitudes. As such, the repeated measures analysis of variance (ANOVA) cannot be adopted for ERN peak/mean amplitudes. Only the nonparametric test could be used. The matrices of the CRN and Pe mean amplitudes in the two experiments were not equivalent (p < 0.01). Thus, the ANOVA could not be adopted for the CRN/Pe mean amplitudes. Only the nonparametric test can be used. The R language based on the F1 LD F1 model was used for the nonparametric ANOVA [33]. As such, the repeated measures ANOVA was used for the CPe mean amplitudes and nonparametric test was used for the other ERP data.

## Results

### Behavioral Results

Table 1 shows the behavioral results. Table 2 shows the relationships between post-error and ERN and the relationships between post-correct and CRN.

**Table 1:** Results of error rates, correct and erroneous RTs, and post-errors and post-corrects for the standard trials.

	Normal	Fatigue
**Error rate**	3 (5)	5 (5)
**RT of correct (ms)**	532.8 (117.2)	567.0 (235.7)
**RT of error (ms)**	511.7 (142.7)	472.6 (146.4)
**Post-error (ms)**	810.5** (344.7)	680.0 **(238.6)
**Post-correct (ms)**	407.3 **(287.8)	331.7 ** (103.9)
**Post-error slowing(ms)**	403.3 (263.6)	340.2 (175.3)

Note: *p < 0.05,

**p < 0.01.

Standard deviations were shown in brackets. Post-error slowing = mean (RT_Post-error) − mean (RT_Post-correct)[53]

**Table 2:** Spearman correlations of post-error (correct) RT, post-error slowing, and error-related ERPs.

		Fz	FC1	Cz	FC2
**Post-error(correct) reaction time**	**ERN Peak**	.093 (.624)	-.096 (.613)	-.118 (.534)	-.140 (.461)
	**ERN Mean amplitude**	.119 (.529)	.041 (.831)	-.056 (.768)	-.038 (.840)
	**Pe Mean amplitude**	-.329 (.076)	-.358 (.052)	-.430* (.018)	-.275 (.142)
	**CRN Mean amplitude**	-.389* (.034)	-.365* (.047)	-.478** (.008)	-.380* (.038)
	**CPe Mean amplitude**	-.529** (.003)	-.401* (.028)	-.412* (.024)	-.356 (.054)
**Post-error slowing**	**ERN Peak**	.026 (.893)	-.146 (.441)	-.245 (.192)	-.207 (.273)
	**ERN Mean amplitude**	.120 (.526)	-.111 (.558)	-.074 (.699)	-.169 (.371)
	**Pe Mean amplitude**	-.224 (.235)	-.188 (.321)	-.313 (.093)	-.217 (.249)
	**CRN Mean amplitude**	-.292 (.117)	-.526** (.003)	-.516** (.004)	-.231 (.220)
	**CPe Mean amplitude**	-.349 (.058)	-.518** (.003)	-.495** (.005)	-.366* (.047)

Note: *p < 0.05,

**p < 0.01.

p values were shown in brackets. CRN and CPe were the correct-related ERPs.

The error rate in the standard trials was larger in the fatigue state with an increasing trend, but without significant difference. Post-error slowing was observed in both experiments (p < 0.001). The Wilcoxon results showed that the RT of post-errors was slower in both experiments. However, post-error slowing was not significantly different between the normal and fatigue experiment groups. Other results, such as error rate, RT of correct, and RT of errors, also showed no significant differences between normal and fatigue states.

Pe/CPe and CRN were negatively correlated with post-error (correct) RT. The post-correct RT and Pe mean amplitudes exhibited a negative correlation in the Cz electrode (*p* < 0.05). The post-correct RT and CRN mean amplitudes were negatively correlated in the Fz (p < 0.05), Cz (p < 0.01), FC1 (p < 0.05), and FC2 (p < 0.05) electrodes. The post-correct RT and CPe mean amplitudes were negatively correlated in the Fz (p < 0.005), Cz (p < 0.05), and FC1 (p < 0.05) electrodes, but not in the FC2 (p < 0.05) electrode. The post-error slowing time and CRN mean amplitudes were negatively correlated in the FC1 (p < 0.01) and Cz (p < 0.005) electrodes, but not in the Fz (p = 0.117) and FC2 (p = 0.220) electrodes. The post-error slowing time and CPe mean amplitudes were negatively correlated in the FC1 (p < 0.005), Cz (p < 0.005), and FC2 (p < 0.05) electrodes, but not in the Fz (p = 0.058) electrode. No significant correlation was observed between the post-correct RT and ERN and between the post-error slowing and ERN.

### Fatigue Questionnaire Results

The results in Table 3 show a higher fatigue level and decreased sustained attention in the fatigue experiment. The sleepiness score (t(28) = −5.054, p < 0.001.) and fatigue subscale (t(28) = −4.703, p < 0.001) and the total score were higher in the fatigue experiment (t(28) = −5.075, p < 0.001). Mental clarity had a low score in the fatigue experiment (t(28) = 4.111, p < 0.001). Decreased sustained attention was obtained in the fatigue experiment, with a low attention score in the normal experiment (t(28) = 4.124, p < 0.001.)

**Table 3:** Mean summary data for fatigue questionnaire subscales.

	Mental clarity	Attention score	Sleepiness	Fatigue	Total score
**Normal**	8.00(1.69)	7.47 (1.81)	2.93(1.94)	3.40(2.06)	10.87 (7.00)
**Fatigue**	5.47(1.68)**	4.87 (1.64) **	6.60(2.05)**	6.93(2.05) **	23.20 (6.29) **

Note: *p < 0.05,

**p < 0.01.

The correlations between the fatigue questionnaire and error-related ERPs are shown in Table 4. The results showed that fatigue state (total score) was significantly and positively associated with ERN mean amplitudes in the Fz (p < 0.005), Cz (p < 0.005), FC1 (p < 0.005), and FC2 (*coefficient* = 0.468, p < 0.01) electrodes. By contrast, fatigue state (total score) was not significantly associated with the Pe amplitudes in the Fz, Cz, FC1, and FC2 electrodes. The sleepiness and fatigue subscales were also significantly and positively associated with ERN mean amplitudes in the Fz, Cz, FC1, and FC2 electrodes. The results indicated a less negative association in the fatigue state. The results of ERN and CRN amplitudes are shown in Table 5.

**Table 4:** Spearman correlations between questionnaire and error-related ERPs (mean amplitudes of ERN and Pe).

		Fz	Cz	FC1	FC2
**ERN**	**Total score**	0.54**(0.002)	0.56**(0.001)	0.53**(0.002)	0.46**(0.009)
	**Attention score**	–0.49**(0.006)	–0.50**(0.004)	-0.48**(0.007)	-0.40*(0.028)
	**Mental clarity**	-0.53**(0.002)	-0.54**(0.002)	-0.54**(0.002)	-0.46**(0.009)
	**Sleepiness**	0.49**(0.005)	0.53**(0.003)	0.51**(0.004)	0.44* (0.013)
	**Fatigue**	0.53**(0.002)	0.56**(0.001)	0.53**(0.002)	0.45*(0.012)
**Pe**	**Total score**	-0.01(0.943)	-0.11(0.555)	0.04(0.827)	0.00(0.962)
	**Attention score**	0.06(0.749)	0.16(0.384)	0.03(0.852)	0.04(0.816)
	**Mental clarity**	0.02(0.886)	0.11(0.555)	-0.00(0.992)	0.00(0.978)
	**Sleepiness**	0.06(0.749)	-0.00(0.984)	0.15(0.422)	0.07(0.680)
	**Fatigue**	-0.04(0.809)	-0.12(0.519)	0.03(0.877)	-0.02(0.879)

Note: *p < 0.05,

**p < 0.01.

p values were shown in the brackets.

**Table 5:** Average ERN peak, ERN/CRN mean, and Pe/CPe mean amplitudes under the normal or fatigue condition.

			Fz	Cz	FC1	FC2
**Normal**	**ERN Peak amplitude**	**Error**	-4.02 (3.99)	-4.77(4.71)	-4.18(4.10)	-3.70(3.84)
		**Correct**	/	/	/	/
	**ERN/CRN Mean amplitude**	**Error**	-2.11(3.07)	-2.75 (3.32)	-2.28(2.94)	-1.86(2.75)
		**Correct**	2.00(2.78)	1.86(2.01)	1.85(2.07)	1.71(1.91)
	**Pe/CPe Mean amplitude**	**Error**	4.62 (3.97)	4.24 (3.56)	4.28(3.26)	4.03(3.33)
		**Correct**	3.44 (3.91)	1.72 (2.16)	2.03(2.54)	2.82(2.76)
**Fatigue**	**ERN Peak amplitude**	**Error**	–1.29 (2.60)*	-1.96 (3.22)	-1.09(2.81)*	-1.32(1.83)*
		**Correct**	/	/	/	/
	**ERN/CRN Mean amplitude**	**Error**	-0.04(1.59)*	-0.34(1.84)*	0.31(1.68)**	0.44(1.24)**
		**Correct**	2.64 (2.77)	2.05 (2.31)	2.30(2.52)	2.70(2.65)
	**Pe/CPe Mean amplitude**	**Error**	3.92 (4.70)	3.83 (3.08)	3.64(3.47)	4.15(3.90)
		**Correct**	4.30 (4.10)	2.54 (2.29)	3.40(3.48)	2.81(2.61)

Note: *p < 0.05,

**p < 0.01.

The table shows the difference between the normal and fatigue groups. Standard deviations were shown in brackets.

Sustained attention state was significantly and negatively associated with ERN mean amplitudes in the Fz (p < 0.01), Cz (p < 0.005), FC1 (p < 0.01), and FC2 (p = 0.05) electrodes, indicating a more negative association in good attention state. Moreover, decreased attention diminished ERN amplitudes. Sustained attention was not significantly associated with the Pe amplitudes. The mental clarity subscale was significantly and negatively associated with ERN mean amplitudes in the Fz, Cz, FC1, and FC2 electrodes.

### ERP Results

**Fig 3:**
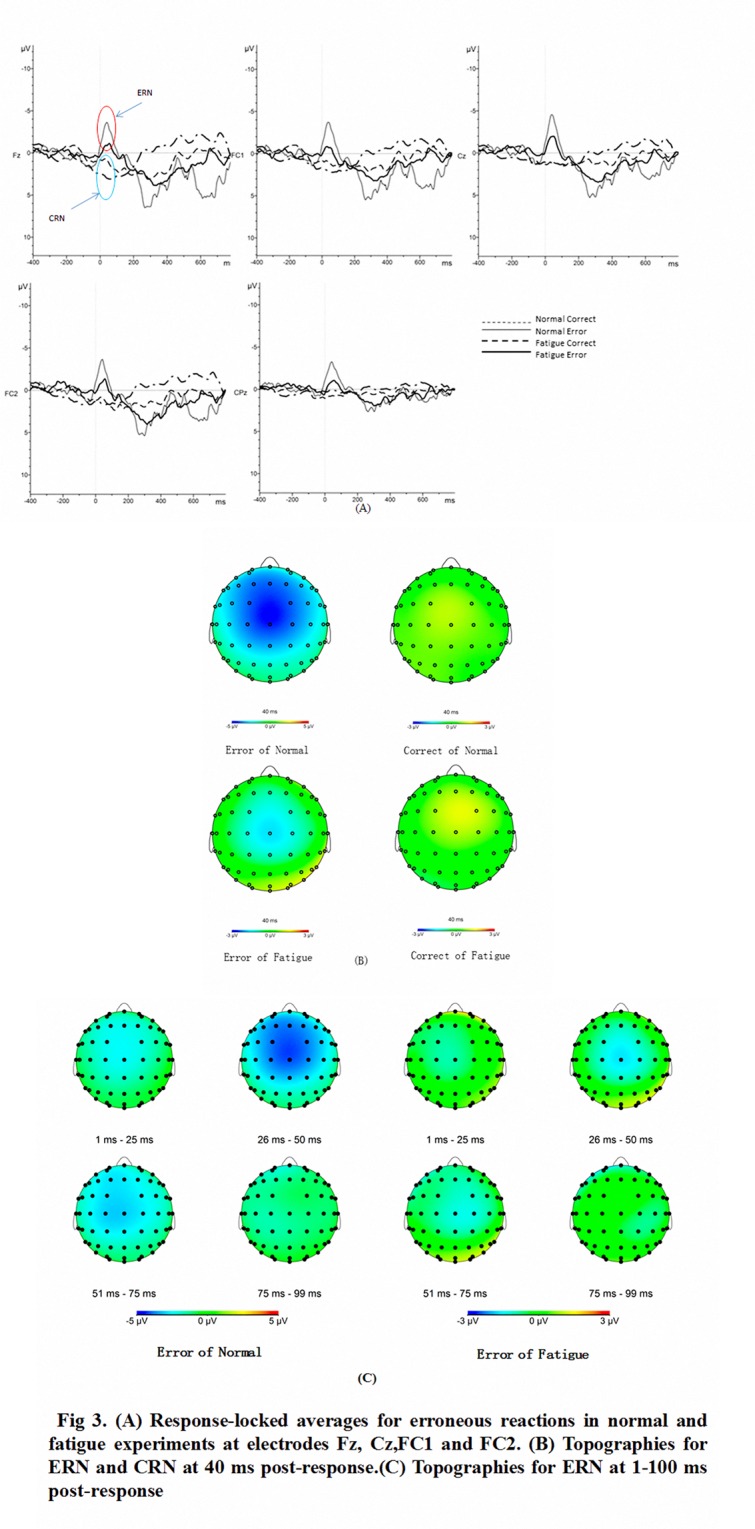
ERN/CRN at electrodes Fz, Cz, FC1, and FC2 in the two experiments. (A) Response-locked averages for erroneous reactions in normal and fatigue experiments at electrodes Fz, Cz,FC1 and FC2. (B) Topographies for ERN and CRN at 40 ms post-response.(C) Topographies for ERN at 1–100 ms post-response.

Fig. 3 shows the response-locked ERPs at electrodes Fz, Cz, FC1, and FC2 in the two experiments with waveforms for erroneous and correct responses. The topographic maps for error-related and correct-related activities at 40 ms post-response are also shown. The group ERN and CRN mean amplitudes are shown in Table 5.

ERN peaks were more negative in the normal experiment than in the fatigue experiment (p < 0.05; without within-subject effect (p = 0.71) and any interaction effect (p = 0.69)). ERN mean amplitudes were more negative in the normal experiment than in the fatigue experiment (p < 0.005; without within-subject effect (p = 0.19) and any interaction effect (p = 0.75)). Meanwhile, the CRN (p = 0.76) and Pe (p = 0.45) mean amplitudes did not differ in the fatigue and normal conditions (p = 0.88; without within-subject effect (p = 0.81) and any interaction effect (p = 0.88, p = 0.60)).

Repeated measures ANOVA showed that the CPe amplitudes were mainly affected by the electrodes (*F*(3,42) = 8.024, p < 0.001), but without any interaction effect. The amplitudes of the Fz electrode were more positive than that of the Cz (p < 0.005) and FC1 (p < 0.05) electrode, without significant difference among other electrodes.

The grand average and topographic maps in Fig. 3 show that ERN amplitudes were more negative in the normal experiment and the active areas were wider. In the fatigue experiment, the active areas were mainly distributed in the FC1, FC2, C1, Cz, C2, CP1, CPz, and CP2 electrodes. By contrast, in the normal experiment, the active areas were extensively distributed in the frontal, fronto-central, and central locations. Fig. 3(C) shows that the activation of ERN was earlier, with a longer duration in the normal condition than in the fatigue condition.

## Discussion

### Mental Fatigue, Attention, and Error Processing

The fatigue questionnaire indicated that the 1 h PVT task induced mental fatigue (with sustained attention and mental clarity decreased, but with sleepiness and fatigue increased) and decreased sustained attention level in the fatigue experiment. The performance of the four-choice RT showed a declining trend in the fatigue condition, but without significant difference. The possible reasons of this finding are as follows: First, many factors may contribute to performance, such as motivation, which may compensate, to some degree, for the negative effects of fatigue on performance. Second, the induced fatigue level might not reach the level at which it would significantly decrease performance.

The degree of ERN amplitude changes was based on the fatigue level. This finding implied that fatigue level contributed to the different degrees of error processing impairment. A higher fatigue level may induce larger ERN amplitude changes. However, low fatigue levels may result in ERN amplitude without any changes, which was likely to occur because fatigue level was insufficient to decrease subject error processing, as observed by Clayson et al. and Wiswede et al. [14,23]. However, if fatigue level extended to the degree that subjects fail to monitor error in any case, ERN would disappear and be unobserved. Fatigue level in this investigation decreased ERN amplitudes, which was similar to the findings of Boksem et al. [20]. The findings provide new evidence to supplement the study of Boksem et al. ERN amplitudes decreased from −9.5 mV in Interval 1 to −3.5 mV in Interval 6 in the study of Boksem et al. [20]. The change level in the study of Boksem et al. was 0.63 ([−9.5 − (−3.5)]/9.5). In this study, ERN amplitudes decreased from −4.77 mV in the normal experiment group to −1.96 mV in the fatigue experiment group at the Cz electrode. The change level is 0.59. This difference may be due to the varying fatigue levels. The relationships between the fatigue questionnaire and ERN amplitudes showed that increased sleepiness and fatigue might diminish ERN amplitudes. The results showed that fatigue was negatively related to error processing. Further investigation should focus on higher fatigue level effects on ERN amplitudes.

The active areas of ERN may be modulated in the fatigue state. Fig. 3(B) shows that ERN amplitudes were more negative in the normal experiment group. Moreover, the active areas were wider. The active areas of ERN were consistent with those of previous studies [8,34]. Impaired error processing in the fatigue state may inhibit nerve activity in some areas.

Mental fatigue can reduce attention, which is important for error processing [35,19,20]. The results of this study showed that sustained attention state was an important factor influencing ERN amplitudes. The correlations between attention and ERN mean amplitudes showed that attention was significantly and negatively associated with ERN amplitudes, which denoted a linear negative relationship between ERN amplitudes and sustained attention state (which means more negative ERN amplitudes with better attention). This finding further proved that the reduced sustained attention state may be the factor that inhibited error processing and decreased ERN amplitudes. The functional diagram shows that the fatigue state decreased sustained attention. Subsequently, decreased sustained attention affected error processing and diminished ERN amplitudes. The degree of change was in line with the attention levels, which was proven by the relationships between attention and ERN amplitudes. This observation provided new evidence to support the conclusion of Boksem et al., which indicated that error processing was impaired in fatigued subjects and decreased attention may be one of the possible influencing factors (see also [36,37]).

The fatigue state could inhibit the activity of the nervous system and decrease cognitive abilities [20,38]. The results of the fatigue questionnaire revealed that sustained attention of the subjects decreased in the fatigue experiment. This finding indicates that lesser attention can be used to finish the task, which may impair error processing and thus may decrease ERN amplitudes. Moreover, with less attention after the response, less error processing was performed by the subjects. The same was true for decreased ERN amplitudes [20]. In other words, the sustained attention state related to the task could inhibit error processing as the task was being undertaken and after the response. This observation was in line with previous studies, which showed increased ERN/CRN with increased attention to response accuracy [2,6,7,39–42]. The relationships between ERN amplitudes and sustained attention presented in this study provided new evidence for this.

### Post-Error Slowing and ERN

Post-error slowing was observed separately in the normal and fatigue experiments groups. Post-error RT was longer than post-correct RT in both experiments. The oral reports of the subjects on errors during the experiment were recorded. The results of the oral reports and post-error slowing showed that the subjects were aware of the error response. This observation was consistent with the results of other previous studies, where post-error slowing was observed for aware errors [43,44].

The relationship between post-error slowing and ERN was not observed, but the relationship between post-error slowing and Pe in the Cz electrode was observed. This observation was inconsistent with certain previous studies that investigated the link between post-error behavioral adaptation effects and ERN [2,8,45–47], but was consistent with some other studies [16,48,49]. Alternatively, as proposed by Notebaert and colleagues, post-error slowing might reflect an unspecific orienting response rather than an adaptive implementation of cognitive control [50].

The post-error slowing of fatigue exhibited a declining trend, but without significant difference in both experiments. The same was true for the error RT. We did not observe a correlation between post-error slowing and ERN, which was consistent with the findings of other studies [2,8,45,46,51]. The oral reports and error results showed that few subjects corrected their incorrect responses and often continued with the response, even when they were reported as errors. As such, post-error slowing may not be positively associated with the increase in accuracy and behavioral adaptation. The observation was consistent with the findings of Danielmeier and Ullsperger, who observed that dissociation between post-error slowing and post-error improvement in accuracy was common [47,51]. This finding implied that different mechanisms might contribute to these effects. Another study revealed that, in the case of post-error slowing, these mechanisms were not necessarily adaptive [52].

### Emotion and Error Processing

Although verbal reports were used, the emotional factor was not considered a strictly controlled variable to examine the relationships. The verbal reports of our experiments revealed that the emotional factor had no effect on the two conditions. Stricter emotion control methods should be used in future research, such as using a standard scale (Profile of Mood States) or synchronously acquiring physiological signals.

## Conclusion

Mental fatigue decreased attention and impaired error processing, but the degree of error processing impairment was related to the fatigue level and attention state. Sustained attention is one of the key capabilities to maintain error processing. The results of the quantitative relationship between sustained attention and ERN amplitudes showed that impaired error processing under the fatigue experiment was most likely affected by decreased sustained attention. Therefore, sustained attention may be one factor that could diminish ERN amplitudes. The current finding will provide a foundation for human error prevention in real tasks by selecting appropriate task-related personnel, who is good at error processing and mobilization of corrective action. The relationships between ERN and post-error slowing were not observed, indicating that post-error slowing may not be positively associated with the increase in accuracy and behavioral adaptation. Novel sustained attention assessment methods and theories should be further studied in future research, which will help us understand the role of sustained attention in error processing.

## Supporting Information

S1 File S1_Dataset. Data of ERP, Questionnaire and Performance.
(RAR)

S2 File S2_highlights. Highlights of the manuscript.
(DOC)

S3 File S3_CERTIFICATE OF ENGLISH EDITING. CERTIFICATE OF ENGLISH EDITING.
(PDF)
