# Supplementary material for: Sustained Attention is Associated with Error Processing Impairment: Evidence from Mental Fatigue Study in Four-Choice Reaction Time Task
Source: PLoS One. 2015 Mar 10;10(3):e0117837. doi: 10.1371/journal.pone.0117837 (PMC4355415; doi:10.1371/journal.pone.0117837)
Supplement: S2 File — Highlights of the manuscript. (DOC) [file pone.0117837.s002.doc]

# Sustained Attention is Associated with Error Processing Impairment: Evidence from Mental Fatigue Study in Four-Choice Reaction Time Task

**Highlights**

We examined the link between sustained attention and error processing used the four-choice reaction time task for the first time.

We examined the quantitative relationship between sustained attention and ERN amplitude.

The degree of error processing impairment was related to fatigue level.

The fatigue group reported higher mental fatigue level and decreased sustained attention.

The fatigue group showed decreased ERN amplitude compared with the normal group.
