# Supplementary material for: Sustained Attention is Associated with Error Processing Impairment: Evidence from Mental Fatigue Study in Four-Choice Reaction Time Task
Source: PLoS One. 2015 Mar 10;10(3):e0117837. doi: 10.1371/journal.pone.0117837 (PMC4355415; doi:10.1371/journal.pone.0117837)
Supplement: S3 File — CERTIFICATE OF ENGLISH EDITING. (PDF) [file pone.0117837.s003.pdf]

EnPapers.Com **CERTIFICATE OF ENGLISH EDITING**

To whom it may concern:

This memo is to certify that the paper titled Sustained Attention is Associated with Error Processing Impairment: Evidence from Mental Fatigue Study in Four-Choice Reaction Time Task has been edited for language by EnPapers, a company dedicated to helping international researchers publish their findings in the best English language journals possible.

Our International paper editing service is performed by a subject expert editor and approved by two senior editors. All our editors are native English speakers.

The certificate is being issued upon the request of the client. If you have any questions, please contact papers@enpapers.com

Signature of the editor representative:

Martin J. Booth
